# Effects of deep neuromuscular block on surgical pleth index-guided remifentanil administration in laparoscopic herniorrhaphy: a prospective randomized trial

**DOI:** 10.1038/s41598-022-23876-5

**Published:** 2022-11-10

**Authors:** In Kyong Yi, Jin-Soo Kim, Hoon Hur, Do-Gyun Han, Ji Eun Kim

**Affiliations:** 1 Department of Anesthesiology and Pain Medicine, Ajou University School of Medicine, 164, World Cup-Ro, Yeongtong-Gu, Suwon, 16499 Korea; 2 Department of Surgery, Ajou University School of Medicine, 164, World Cup-Ro, Yeongtong-Gu, Suwon, 16499 Korea

**Keywords:** Health care, Medical research

## Abstract

Deep neuromuscular block (NMB) has been increasingly utilized, but its role in reducing intraoperative opioid requirement has yet to be investigated. Surgical pleth index (SPI) quantifies nociception. We investigated the effects of deep NMB on SPI-guided remifentanil administration in laparoscopic herniorrhaphy. Total 128 patients undergoing laparoscopic inguinal herniorrhaphy were randomly allocated to two groups of NMB: deep (n = 64) and moderate (n = 64). The remifentanil dose was assessed during intubation, from skin incision until CO_2_ insertion, and pneumoperitoneum. Mean infusion rate of remifentanil during pneumoperitoneum was higher in moderate NMB group than in deep NMB group (0.103 [0.075–0.143] µg/kg/min vs. 0.073 [0.056–0.097] µg/kg/min, *p* < 0.001). Consequently, median infusion rate of remifentanil during anesthesia was higher in moderate NMB group (0.076 [0.096–0.067] µg/kg/min vs. 0.067 [0.084–0.058] µg/kg/min, *p* = 0.016). The duration of post-anesthesia care unit stay was longer in the moderate NMB group (40 [30–40] min vs. 30 [30–40] min, *p* = 0.045). In conclusion, deep NMB reduced the remifentanil requirement compared with moderate NMB in SPI-guided anesthesia for laparoscopic herniorrhaphy.

## Introduction

Laparoscopy is routinely used in general surgery due to less inflammation, immunosuppression, bleeding, postoperative pain, and rapid recovery compared with open laparotomy^1,2^. However, laparoscopic surgery requires elevated intra-abdominal pressure (IAP) and steep changes in position, which lead to pathophysiological aberrations, which are a challenge to anesthesiologists^3^. To reduce the risk of these complications, the international guideline recommends the use of the lowest IAP possible; however, it can impair the quality of the surgical field^4^.

Since the introduction of sugammadex, the potential advantages of deep NMB in laparoscopic surgery have been extensively investigated. Deep neuromuscular block (NMB) lowers IAP and improves surgical space at a time, thus enhancing the surgical outcome^5^. Besides, deep NMB reduces laparoscopy-related lung injury and postoperative pain^5–7^.

Surgical pleth index (SPI) can be used to detect cardiovascular changes resulting from nociception-induced sympatho-vagal imbalance and quantify the nociception for titration of analgesic administration^8^. SPI-guided anesthesia is associated with clinical advantages compared with standard analgesic practice based on clinical parameters; including earlier injection of sufentanil bolus, stabler modulation of CO_2_ insufflation-induced sympathetic activation, faster recovery and reduced remifentanil dose^9–12^. These advantages reinforce the role of SPI in anesthetic management^13^.

Studies comparing deep NMB with moderate NMB during laparoscopic surgery utilized similar opioid doses^6,14–16^. In there, all opioids were administered via standard analgesic practice. Currently, the superior effects of deep NMB during laparoscopic surgery are disputed, while the benefits of deep NMB in intraoperative opioid treatment have rarely been investigated clinically^17,18^. Therefore, we hypothesized that deep NMB reduces the need for remifentanil compared with moderate NMB, when guided by SPI. The aim of this study was to investigate the effects of deep NMB on remifentanil requirements in patients undergoing SPI-guided anesthesia for laparoscopic inguinal herniorrhaphy.

## Methods

### Patients

This prospective, double-blind, randomized controlled trial was approved by institutional review board of Ajou University Hospital (AJIRB-MED-THE-19-056, 9 April 2019) and registered with ClinicalTrials.gov (NCT04022733, 17 July 2019). Written informed consent was obtained from eligible patients with American Society of Anesthesiologists (ASA) physical status classification I–III, aged 19–85 years, and undergoing elective laparoscopic inguinal herniorrhaphy from October 2019 to August 2021. We excluded patients with arrythmia, hyperbilirubinemia, chronic pain, opioid abuse, infection, and the possibility of conversion to open herniorrhaphy. Peripheral vascular disease was excluded but diabetes was included.

### Interventions

A total of 134 participants were randomly assigned to 2 groups (deep and moderate) depending on the depth of NMB using a computer-generated random table (http://www.random.org). In the deep NMB group (n = 67), NMB was maintained as post-tetanic count (PTC) 1–2 during surgery and reversed using sugammadex 4 mg/kg based on actual body weight after completion of surgery. In the moderate NMB group (n = 67), NMB was maintained as a train-of-four (TOF) count 1–2 and reversed with neostigmine neostigmine 50 μg/kg and glycopyrrolate 10 μg/kg. NMB was monitored using kinemyography (MechanoSensor™; GE healthcare, Chicago, IL, USA) administered to the adductor pollicis muscle. An assistant who was not involved in the trial performed the randomization in a 1:1 ratio and concealed the allocation sequence in opaque, sealed envelopes. The study interventions including NMB titration and administration of reversal agent were carried out by an independent investigator (J.E.K.) who did not participate in the outcome assessment. The other investigators and the patients were blinded to the group assignment.

Trendelenburg position was set to 30°. Pneumoperitoneum was controlled by limiting CO_2_ insufflator, and IAP was maintained at 12 mmHg in both groups.

### Anesthesia

Two investigators (I.K.Y. and D.-G.H) managed the anesthesia according to a protocol. Without premedication, patients were monitored via electrocardiography, non-invasive arterial pressure measurement, pulse oximetry, and SedLine® (Masimo, Irvine, CA, USA). A pulse oximeter sensor for SPI (GE healthcare, Chicago, IL, USA) was applied to index finger of contralateral side to the arm with an arterial pressure cuff. SPI is computed using an algorithm (1) that combines normalized heartbeat interval (HBI_norm_) and normalized photoplethysmographic pulse wave amplitude (PPGA_norm_)^19^:

1 $$ {\text{SPI}} = {1}00{-}\left( {0.{33} \times {\text{HBI}}_{{{\text{norm}}}} } \right) + \left( {0.{67} \times {\text{PPGA}}_{{{\text{norm}}}} } \right) $$

A balanced anesthesia was implemented with sevoflurane and remifentanil (Ultiva; GlaxoSmithKline, Brentford, UK) via target-controlled infusion based on Minto’s pharmacokinetics. Following pre-oxygenation, anesthesia was induced with intravenous (IV) propofol 2 mg/kg and remifentanil of 3–5 ng/mL as target concentration. MechanoSensor™ was calibrated and stabilized (< 5% variation in the TOF ratios) after the loss of consciousness. Subsequently, IV rocuronium bromide 0.6 mg/kg was administered. Following confirmation of relaxation, patients were intubated with a videolaryngoscope. Mechanical ventilation was achieved with a tidal volume of 6–8 mL/kg, positive end-expiratory pressure of 5 cmH_2_O and an inspired oxygen fraction 0.5. Respiratory rate was changed for an end-tidal carbon dioxide tension of 30–40 mmHg. Normal saline or plasmalyte was infused at a rate of 6 mL/kg/hr.

Anesthetic depth was maintained at a Sedline® patient state index (PSi) of 25–50 by adjusting the end-tidal concentration of sevoflurane. Rocuronium 0.3–0.4 mg/kg/h was continuously infused and titrated according to the group assignment until the end of the fascial suturing (by J.E.K.). The remifentanil dose was adjusted to maintain an SPI range of 20–50. To manage the hypotension associated with a mean arterial pressure (MAP) < 60 mmHg, a bolus of ephedrine 4 mg was primarily administered along with an infusion of norepinephrine as needed. An IV propacetamol 1 g was given at the end of the surgery. NMB was reversed according to the group assignment (by J.E.K.), and extubation was performed after confirming the TOF ratio > 0.9. Patients were then transferred to a post-anesthesia care unit (PACU).

### Data collection

The investigators (I.K.Y. and D.-G.H) recorded the time and remifentanil dose during the surgery. Since the dynamic conditions such as endotracheal intubation or skin incision possibly affect the remifentanil dose requirement during deep NMB, each remifentanil dose was assessed three times: during intubation, time from skin incision until CO_2_ insertion, and time from CO_2_ insertion until the removal of laparoscope (pneumoperitoneum). In addition, the remifentanil concentration was maintained at 0 ng/mL after intubation and then increased to 3.0 ng/mL immediately before skin incision. Infusion rate of remifentanil (µg/kg/min) was calculated by adjusting total doses of remifentanil using body weight and infusion time.

During surgery, SPI, PSi, and hemodynamic parameters such as heart rate (HR) and MAP were recorded at the following four time points: before induction (T0), 10 min after induction (T1), 20 min after CO_2_ insertion (T2), and the removal of laparoscopy (T3). In the PACU, the investigator (D.-G.H) evaluated the recovery data at 30 min after the end of surgery. Nausea (1 = none, 2 = mild, 3 = moderate, and 4 = severe), vomiting, and pain scores (11-point numerical rating scale, 0 = no pain, 10 = worst pain) were assessed. IV ramosetron 3 mg was administered as a rescue antiemetics in the event of nausea ≥ 3, and fentanyl 0.5 μg/kg was used as a rescue analgesia when the pain score was ≥ 5.

### Sample size calculation

The primary outcome was the infusion rate of remifentanil during pneumoperitoneum. Based on the findings of a previous study^20^, we considered a difference in remifentanil infusion rate > 0.032 µg/kg/min (16% of mean infusion rate of remifentanil in moderate NMB under SPI-guided anesthesia for laparoscopic surgery [mean infusion rate of 0.192 (SD 0.064) µg/kg/min]) as clinically relevant. Based on a significance level of 5% and statistical power of 80%, each group required 63 subjects for analysis. We enrolled a total of 134 patients to compensate for dropouts and observational variation.

### Statistical analysis

Statistical analyses were conducted using SPSS software version 25.0 (IBM Corp., Armonk, NY, USA). Data are presented as mean ± SD, median (interquartile range), and number of patients. The normality of distribution was tested using the Kolmogorov–Smirnov. Continuous data were compared using a two-tailed t test when normally distributed; however, Mann–Whitney *U* test was used for non-normally distributed data. Categorical data were compared using the Chi-square test or Fisher’s exact test, as appropriate. Intergroup comparisons for repetitively measured data were performed by unpaired *t*-test with Bonferroni correction. There was no multiplicity adjustment made for multiple comparisons. All tests were two-sided, and *p* values < 0.05 were considered statistically significant.

### Institutional review board statement

The study was conducted according to the guidelines of the Declaration of Helsinki, and approved by the Institutional Review Board of Ajou Hospital (AJIRB-MED-THE-19-056, April 2019).

### Informed consent

Informed consent was obtained from all subjects involved in the study.

## Results

### Study population

**Figure 1:**
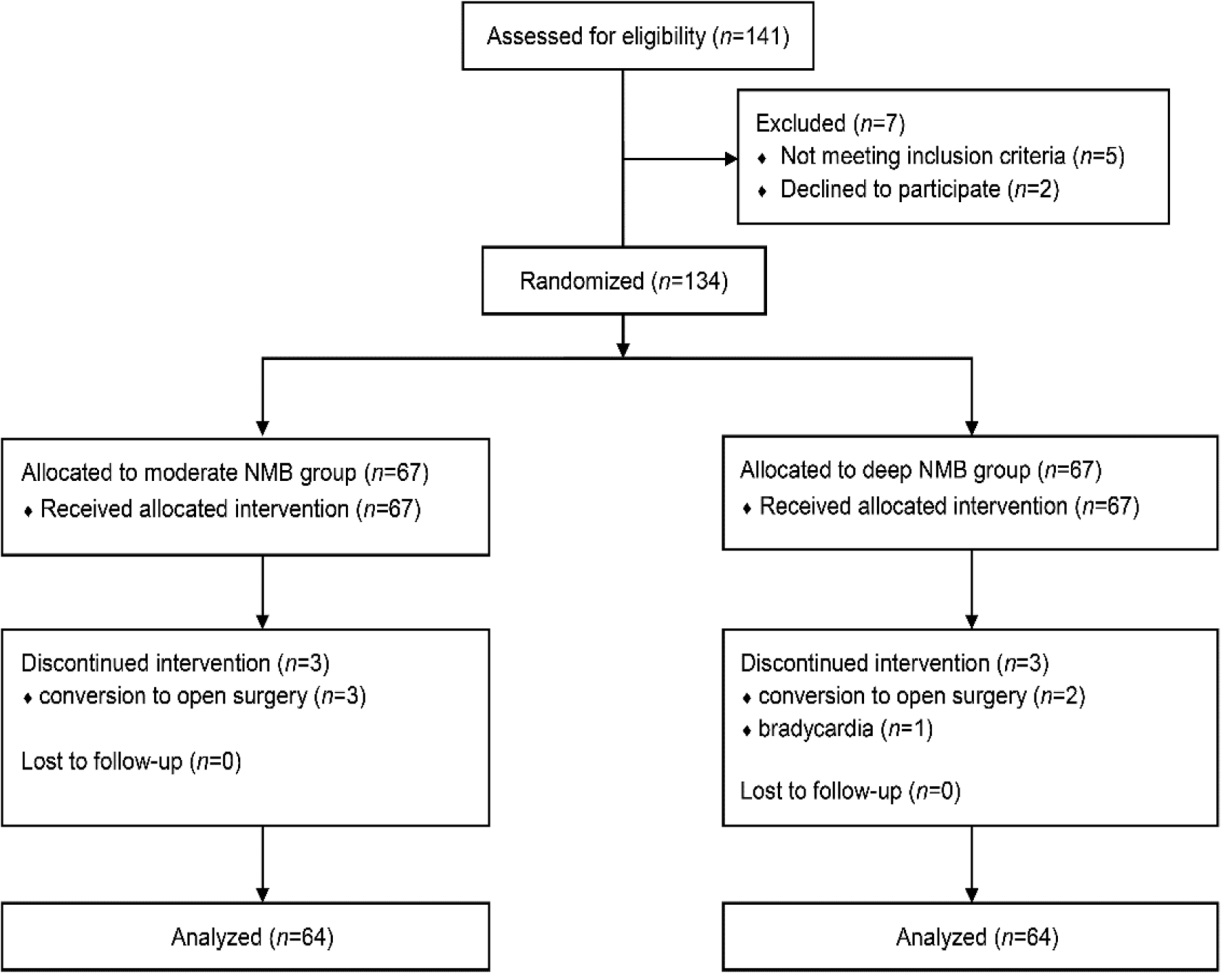
The CONSORT flow diagram of patient enrollments. NMB, neuromuscular block.

A total of 134 participants were enrolled and randomized. However, 6 participants dropped out of the study due to conversion to open herniorrhaphy (n = 5) and bradycardia during anesthetic induction (n = 1) (Fig. 1).

### Baseline characteristics

Patient characteristics and intraoperative data were comparable between two groups, except for the total dose of rocuronium used during the anesthesia (Table 1). Hemodynamic parameters, SPI and PSi were adequately maintained throughout the study period (Table 2). Although MAP, SPI and PSi did not differ between two groups, HR in the deep NMB group was higher than that in the moderate NMB group at T2 (*p*_*adjusted*_ = 0.040). Pneumoperitoneum was adequately maintained in all patients without changing the IAP.

**Table 1:** Patient characteristics and operative data. Values are presented as the mean ± standard deviation, median (interquartile range), and number of patients. NMB, neuromuscular block; BMI, body mass index; ASA, American Society of Anesthesiologists.

	Moderate NMB (n = 64)	Deep NMB (n = 64)	*p* value
Age (yr)	63 (54–70)	65 (51–75)	0.663
Sex (male/female)	58/6	59/5	0.752
Height (cm)	168 ± 8	166 ± 6	0.132
Weight (kg)	66 ± 11	66 ± 10	0.928
BMI (kg/m^2^)	23.6 (21.7–25.6)	23 (21.5–25.7)	0.724
ASA physical status (I/II/III)	18/41/5	22/33/9	0.300
Diabetes	9	12	0.474
Inguinal hernia (unilateral/bilateral)	59/5	57/7	0.763
**Intraoperative data**			
Fluid (mL)	450 (350–550)	475 (400–600)	0.706
Bleeding (mL)	10 (10–20)	10 (10–20)	0.383
Total dose of rocuronium (mg)	45 (40–49)	75 (65–75)	< 0.001^†^
Intraoperative medications*			
Norepinephrine	4	3	> 0.999
Ephedrine	38	31	0.215
Isosorbide dinitrate	1	2	> 0.999
**Durations**			
Surgery (min)	37.5 (30–47.5)	40 (30–55)	0.131
Anesthesia (min)	72.5 (65–87.5)	75 (65–85)	0.227

*Number of patients receiving vasoactive drugs. ^†^*p *value < 0.05 between-group comparison.

**Table 2:** Hemodynamic parameters and data associated with anesthesia.

	Moderate NMB (n = 64)	Deep NMB (n = 64)	Adjusted *p* value
**Heart rate (beats/min)**			
T0	70 ± 12	71 ± 13	> 0.999
T1	67 ± 13	70 ± 12	0.532
T2	61 ± 10	66 ± 11	0.040^†^
T3	60 ± 10	64 ± 10	0.128
**Mean arterial pressure (mmHg)**			
T0	106 ± 13	107 ± 17	> 0.999
T1	80 ± 14	78 ± 12	> 0.999
T2	86 ± 13	87 ± 13	> 0.999
T3	83 ± 12	84 ± 11	> 0.999
**Surgical pleth index (SPI)**			
T0	73 ± 15	73 ± 16	> 0.999
T1	33 ± 11	33 ± 10	> 0.999
T2	37 ± 12	35 ± 11	> 0.999
T3	36 ± 15	35 ± 13	> 0.999
**Patient state index (PSi)**			
T0	95 ± 5	94 ± 5	> 0.999
T1	37 ± 7	38 ± 8	> 0.999
T2	34 ± 7	33 ± 7	> 0.999
T3	34 ± 6	33 ± 7	> 0.999

Values are presented as the mean ± standard deviation. NMB, neuromuscular block. T0, before induction; T1, 10 min after induction; T2, 20 min after CO_2_ insertion; T3, the removal of laparoscopy. ^†^*p *value < 0.05 between-group comparison.

### Remifentanil doses

Remifentanil doses administered at each time point are presented in Table 3. No significant differences were detected in total doses or infusion rate of remifentanil during intubation and during time from skin incision until CO_2_ insertion (*p* = 0.770, *p* = 0.708 and *p* = 0.571, respectively). In addition, the total dose of remifentanil during pneumoperitoneum was comparable between the two groups (*p* = 0.074). However, when adjusted for body weight and infusion time, the median infusion rate of remifentanil during pneumoperitoneum was significantly higher in the moderate NMB group than in the deep NMB group (0.103 [0.075–0.143] µg/kg/min vs. 0.073 [0.056–0.097] µg/kg/min, *p* < 0.001). Consequently, the median infusion rate of remifentanil during anesthesia also was significantly higher in the moderate NMB group than in the deep NMB group (0.076 [0.096–0.067] µg/kg/min vs. 0.067 [0.084–0.058] µg/kg/min, *p* = 0.016).

**Table 3:** Remifentanil doses.

	Moderate NMB (n = 64)	Deep NMB (n = 64)	*p* value
**During intubation**			
Total dose of remifentanil (µg)	100.7 (22.3)	101.9 (25.9)	0.770
**During time from skin incision until CO**_**2**_** insertion**			
Duration (min)	2 (2–4)	2 (2–3)	0.916
Total dose of remifentanil (µg)	52.5 (35.5–70.6)	53 (42–66)	0.708
Infusion rate of remifentanil (µg/kg/min)	0.276 (0.180–0.551)	0.336 (0.222–0.456)	0.571
During pneumoperitoneum*			
Duration (min)	26 (20–35)	30 (22–43.5)	0.102
Total dose of remifentanil (µg)	179 (128.5–258.5)	142.5 (103.5–241)	0.074
Infusion rate of remifentanil (µg/kg/min)	0.103 (0.075–0.143)	0.073 (0.056–0.097)	< 0.001^†^
**During anesthesia**			
Total dose of remifentanil (µg)	364.5 (288.5–450)	352.5 (262–455.5)	0.555
Infusion rate of remifentanil (µg/kg/min)	0.076 (0.096–0.067)	0.067 (0.084–0.058)	0.016^†^

Values are presented as the mean ± standard deviation and the median (interquartile range). NMB, neuromuscular block; CO_2_, carbon dioxide. *defined as the time from CO_2_ insertion until the removal of endoscopy. ^†^*p *value < 0.05 between-group comparison.

### Recovery data

Recovery data at PACU are presented in Table 4. Although no differences were found in pain score, nausea, vomiting, the numbers of patients receiving analgesics or antiemetics, and the duration of hospital stay, the duration of PACU stay was significantly longer in the moderate NMB group than in the deep NMB group (40 [30–40] min vs. 30 [30–40] min, *p* = 0.045). Postoperatively, none of the patients exhibited any complications regarding deep NMB or sugammadex use.

**Table 4:** Recovery data.

	Moderate NMB (n = 64)	Deep NMB (n = 62)*	*p* value
Pain score (0–10)	4 (3–6)	4 (3–5)	0.279
Nausea (1/2/3/4)	17/43/3/1	15/46/1/0	0.484
Vomiting	1	0	> 0.999
Number of patients receiving analgesics	26	25	0.972
Number of patients receiving antiemetics	1	0	> 0.999
Duration of PACU stay (min)	40 (30–40)	30 (30–40)	0.045^†^
Duration of hospital stay (day)	3 (2–3)	3 (2–3)	0.657

Values are presented as the median (interquartile range) or number of subjects. NMB, neuromuscular block; PACU, post-anesthesia care unit. *2 patients transferred to intensive care unit in deep NMB group. ^†^*p *value < 0.05 between-group comparison.

## Discussion

This study is the first that SPI-guided anesthesia was used to evaluate the effects of deep NMB on remifentanil required during surgery. The deep NMB significantly reduced the intraoperative remifentanil requirements compared with the moderate NMB in patients undergoing SPI-guided anesthesia for laparoscopic inguinal herniorrhaphy. In addition, the duration of PACU stay was significantly shorter in the deep NMB group, despite similar pain scores and rescue analgesic treatment.

A high degree of muscle relaxation is needed for more complex laparoscopic procedures. Sugammadex, which antagonizes rocuronium at any level of NMB, can be used to prolong deep NMB right until the very end of the surgery^21^. Evidences support the routine use of deep NMB during laparoscopic surgery^5,22^. However, it is difficult to establish whether specific outcomes can be attributed to the effects of deep NMB or low IAP (< 12 mmHg). Also, there is a possibility that the presence of a deep NMB could influence on lowering IAP, thus different IAPs across the enrolled studies constituted a limitation^22^. Given the detrimental effects of pneumoperitoneum on intra-abdominal organ circulation and cardiopulmonary function, a low IAP is clinically advantageous compared with standard IAP (12 mmHg)^23,24^. Therefore, a distinction between the effects of low IAP and deep NMB is essential. Martini et al. reported that deep NMB improves the quality of surgical conditions compared with moderate NMB during laparoscopy without a cardiorespiratory compromise under identical retroperitoneal pressure conditions^16^. A subsequent meta-analysis showed that deep NMB improved the surgical space at low and high IAP^5^. Therefore, our study was designed to analyze the effects of NMB (deep vs. moderate) on surgical conditions under identical and standard IAP levels (12 mmHg). In addition, SPI reduced the levels of intraoperative analgesia compared with standard clinical practice, which was found in sevoflurane anesthesia but not in propofol anesthesia^12^. In this regard, we used sevoflurane and remifentanil anesthesia in our study.

The reduced need for remifentanil in our study can be explained as follows. First, deep NMB might reduce surgical stress compared with moderate NMB in our study. Stress response to surgical trauma activates the sympathoadrenal, endocrine and immunologic response^25^. Laparoscopic hernia repair is associated with less tissue injury than open approach, and thus decreases the inflammatory stress response^26^. However, hormonal stress response (catecholamines and cortisol) might not be altered significantly than in open hernia repair, because the stimuli for stress response originate in the visceral and peritoneal afferent nerve as well as in the abdominal wall^25,27^. Further, the pneumoperitoneum significantly decreased the oxygenation and perfusion in abdominal organ, which is associated with the increased stress response^24,27–30^. Consequently, the sympathetic activation results in cardiovascular effects such as tachycardia and hypertension, thereby increasing the SPI values. In addition, remifentanil was found to suppress the stress response notably in various conditions^31–33^. Tools such as SPI correlate effect-site concentrations of remifentanil better than other clinical parameters^13^. Since both groups were operated under an identical and standard IAP, deep NMB appears to reduce the surgical stress in our study entirely. In a previous study, Koo et al.^33^ investigated the inflammatory stress response (e.g., interleukins, tumor necrosis factor-α, and C-reactive protein) in relation to the depth of NMB in laparoscopic gastrectomy, and found no differences between the groups. Although the incidence of unwanted events such as spontaneous breathing was lower in the deep NMB group, they infused the remifentanil using vital signs as a guide, not SPI guide, and did not even measure a remifentanil consumption^34^. A further study is needed to evaluate hormonal stress response in deep and moderate NMB under SPI guidance.

Second, it might be explained by the effects of rocuronium on vascular tone partly. Non-depolarizing NMB agents act as antagonists in nicotinic receptors at the neuromuscular junction, but also bind to muscarinic cholinergic receptors on vascular smooth muscle^35^. In studies investigating the direct effects of muscle relaxants on vascular smooth muscle contraction and relaxation, the relaxation effects increased in the following order: pancuronium < rocuronium < vecuronium^36,37^. Clinically, compared with pancuronium, rocuronium decreased the HR at 5 min after injection in patients undergoing cardiac surgery, combined with morphine treatment^38^. However, rocuronium induced a mild increase in HR and decreased MAP in a study enrolled the various surgeries^39^. Based on a mild vagolytic action, the higher dose of rocuronium used to maintain the deep NMB would reduce the need for remifentanil in our study. Although there were no significant decreases in MAP despite higher HR at T2, the vasodilative property of rocuronium might be manifested because the coefficient of PPGA is about twice as large as that of HBI, indicating PPGA is more important than HBI when calculating the SPI value^19^.

Theoretically, deep NMB enables maximum stretching of abdominal wall muscle during laparoscopy, and could reduce pressure-induced postoperative pain. Studies investigating the use of deep NMB in laparoscopy-related pain show conflicting results. In 2017, a meta-analysis reported a significant reduction of early postoperative pain after deep NMB, including 5 studies using low or standard IAPs or altered pressure, underscoring the need for separation between the effects of deep NMB and low IAP^5^. Other studies found that deep NMB did not reduce the intensity of pain at PACU compared with moderate NMB under identical and standard IAP in laparoscopic cholecystectomy, laparoscopic and robotic gastrectomy^40–42^. Similarly, the pain scores at PACU did not differ in our study under identical IAPs (12 mmHg). In contrast, deep NMB reduced postoperative pain compared to moderate NMB in bariatric surgery under identical but elevated IAP (18 mmHg)^6^. A further study investigating the effects of deep NMB on postoperative pain in invasive surgery (e.g., laparotomy) is needed.

Remifentanil, an ultra-short acting opioid, has been widely used as an infusion for induction of general anesthesia. However, excessive intraoperative administration of remifentanil delays recovery from anesthesia and is associated with acute opioid tolerance and opioid-induced hyperalgesia^43,44^. Remifentanil infusion rates of > 0.25 µg/kg/min and > 0.2 µg/kg/min were associated with tolerance and hyperalgesia, respectively^44^. The infusion rates of 0.076 µg/kg/min and 0.067 µg/kg/min in moderate and deep NMB groups in our study might not affect pain score and analgesic use at PACU due to remifentanil-related tolerance or hyperalgesia. The low infusion rates might be attributed not only to minimal invasiveness of laparoscopy, but also the advantages of SPI over standard clinical practice in intraoperative analgesia^12^.

The duration of PACU stay was significantly shorter in the deep NMB group in our study. In the absence of differences in other outcomes at PACU, the shorter duration and early postoperative discharge may be attributed to sugammadex^45^. Rapid discharge to the surgical ward may be explained by sugammadex-related reduced respiratory events, complete reversal from NMB, faster arousal and neostigmine-related adverse effects associated with muscarinic antagonists^45^. Despite the higher dose of rocuronium in deep NMB, the reduced time of PACU stay in our study suggests the superiority of sugammadex compared with conventional agents in terms of recovery.

The study has several limitations. First, the effects of opioid vary in quality and quantity according to age. Therefore, future studies involving adults and elderly populations are needed. Second, the comparison between deep NMB plus low IAP and moderate NMB plus standard IAP can highlight the enhanced postoperative outcomes as suggested in an editorial^46^. Third, the depth of NMB could have been seen unintentionally by the two investigators participating in the anesthesia and outcome assessment, although all interventions were conducted only by an independent investigator (J.E.K.). Fourth, it is better to administer sugammadex 2 mg/kg as a reversal agent to patients in the moderate NMB group on par with those in the deep NMB group, instead of neostigmine and glycopyrrolate, to confirm the unique effect of NMB depth on recovery in PACU and to overcome the ethical concerns associated with the best treatment options available for the patient. Lastly, the updated 2021 guidelines of Standards of Monitoring during Anesthesia and Recovery recommend the use of “automated electronic anesthetic record systems” for accuracy of information^47,48^. A further study based on intraoperative electronic hemodynamic data, not a specific time points, is warranted to demonstrate the vasodilative effects of rocuronium.

## Conclusions

Deep NMB significantly reduced the remifentanil requirements compared with moderate NMB in patients undergoing SPI-guided anesthesia for laparoscopic inguinal herniorrhaphy. In addition, the deep NMB accelerated the PACU discharge.

## Data Availability

The data presented in this study are available on request from the corresponding author. The data are not publicly available due to privacy.
